# The Performance of ICDAS-II Using Low-Powered Magnification with Light-Emitting Diode Headlight and Alternating Current Impedance Spectroscopy Device for Detection of Occlusal Caries on Primary Molars

**DOI:** 10.1155/2013/276070

**Published:** 2013-07-14

**Authors:** Timucin Ari, Nilgun Ari

**Affiliations:** Western University Schulich School of Medicine and Dentistry, Dental Sciences Building, Room 1017, London, ON, Canada N6A 5C1

## Abstract

Early detection of occlusal caries in children is challenging for the dentists, because of the morphology of pit and fissures. The aim of this study was to compare in vitro the diagnostic performance of low-powered magnification with light-emitting diode headlight (LPMLED) using ICDAS-II criteria and AC Impedance Spectroscopy (ACIS) device, on occlusal surfaces of primary molars. The occlusal surfaces of 18 extracted primary molars were examined blindly by two examiners. The teeth were sectioned and examined under light microscopy using Downer's histological criteria as gold standard. Good to excellent inter- and intraexaminer reproducibility, higher sensitivity, specificity, and AUC values were achieved by LPMLED at D1 threshold. Also the relationship between histology and LPMLED was statistically significant. In conclusion visual aids have the potential to improve the performance of early caries detection and clinical diagnostics in children. Despite its potential, ACIS device should be considered as an adjunct method in detecting caries on primary teeth.

## 1. Introduction

It is well established that caries levels in industrialized nations have decreased over the last few decades with the greatest reductions occurring on the smooth and approximal surfaces [1–4]. Because of the complex occlusal anatomy, more sensitive and reproducible diagnostic tools for precise caries detection in children are needed [5]. Visual examination still is the most commonly used method for detecting dental caries, but various studies showed problems for sensitivity and reproducibility problems [6–8]. A standardized scoring system, International Caries Detection and Assessment System (ICDAS-II), has been developed for clinical practice and research to overcome these problems [9]. A complimentary approach to visual examination is to use visual aids such as low-powered magnification (dental loupes) and special headlights mounted on them. These visual aids became popular among dentists to improve precision of visual examination and for ergonomic reasons [10, 11]. Advances in caries research led novel technologies to help dentists in the diagnosis of early lesions. ACIS device (CarieScan PRO, Dundee, Scotland) is one of the recent examples of the novel technologies. This device relies on the application of a small alternating electrical signal (undetectable by the patient) through the tooth while monitoring the response at the sensor. By changing frequency of the applied signal, a spectrum is captured which provides valuable insights into the physical and chemical properties of the tooth. The result is displayed on the LCD screen and the color LED display that enables dental professionals to evaluate the depth of the carious lesion. Pediatric dentistry, with its small operating field and its demands for manual skills and precision, is particularly suited to the use of novel technologies and visual aids. 

Therefore the aim of this study was to compare in vitro the diagnostic performance of low-powered magnification (2.5x) with mounted LED headlight illumination using ICDAS-II criteria and AC Impedance Spectroscopy device, on occlusal surfaces of primary molars. 

## 2. Materials and Methods

Prior to undertaking the study, ethical approval was granted by Western University Research Ethics Board for Health Sciences Research (File no. 101093). Eighteen recently extracted second primary molars (*n* = 18) were selected for this in vitro study. Extracted teeth were kept in 10% neutral buffered formalin immediately following extraction. Only teeth with sound to incipient lesions were selected; teeth with occlusal restorations, occlusal fissure sealants, and hypoplastic pits were excluded from this study. Prior to examinations, each tooth surface was cleaned with pumice and water slurry to remove any debris and rinsed thoroughly in sterile water. The teeth were mounted to impression putty (VP Mix Putty, Henry Schein Inc., USA) in order to mimic intraoral anatomical position for mixed dentition.

The details of each score for ICDAS-II examination and ACIS device instructions were discussed. Examiners were calibrated by a training exercise on both techniques followed by discussion to consensus of any uncertainties. 

In order to assess intra- and interexaminer reproducibility, 15 primary molars (7 primary 1st molars and 8 primary 2nd molars) that were not included in the present study were examined on two separate occasions with two weeks interval by both examiners. All examinations were conducted under standard conditions in dental surgery, with conventional dental light (A-dec, OR, USA) and 3 : 1 syringe. The teeth were positioned 40 cm to examiners' eyes and kept wet during the examinations unless when dried for ICDAS-II examination. One site on each tooth was selected on the occlusal surface, and examiners were guided by black and white photographs printed on draft-quality paper containing a dot on the test site to allow the precise assessment of the same area. The examinations were first carried out with custom made dental loupe (2.5x magnification) with mounted LED headlight (Univet Optical Technologies, Italy) and then AC Impedance Spectroscopy device (CarieScan PRO, Dundee, Scotland) on separate occasions. 

After all examinations were completed, the roots of the teeth were resected just apical to the cementum-enamel junction prior to histological examination. A marker was placed on the mesial cervical area of each tooth, and nail varnish was applied to this mesial groove to aid identification of tooth surfaces and therefore orientation after sectioning. To obtain the histological sections, each tooth was immersed in orthodontic resin (Caulk Orthodontic Resin, Dentsply, USA) and allowed to set into blocks (18 individual blocks), with approximately 1 cm to one side. Each mounted block was then serially sectioned in a longitudinal buccolingual direction with a water-cooled diamond disc on a thin sectioning machine (Gillings-Hamco, NY, USA). Each section was approximately 350 micron thick, and based on visible caries location the cuts were done approximately every 200 microns. The sections were separated from the block and numbered for examination. After sectioning the grooves and artifacts left by the diamond disc were polished with a fine-grained paper coated with 600, 1200, and 2400 grade aluminum oxide (Al_2_O_3_). In total 7–10 sections were produced per crown and 1–4 sections were available to view for each investigation site. Histological sections were examined under a Nikon SMZ-1500 stereomicroscope (Nikon Instruments, Inc., Melville, NY) and digital images were captured with incident light at ×16 magnification. 

All histological sections for each tooth were assessed by both examiners who were blind to each other according to five-point scale Downer histological classification system (Table 1) [12]. Caries extent was based upon colour and structural changes in enamel and dentine, with emphasis being placed on differentiating carious changes from protective changes of the pulp-dentine complex, such as tubular sclerosis and reactionary dentine formation. A histological score was given to each section and the deepest score section was taken as the definitive for further analysis. Where there was disagreement, two examiners reviewed the sections again and new examinations were performed until a consensus decision was reached.

## 3. Data Management and Statistical Evaluation

ICDAS-II scores using LPMLED and ACIS device readings and histology scores were recorded on special sheets and transferred to an Excel table. The statistical analysis was performed using MedCalc v.9.0.1.1 statistical package (MedCalc Software, Mariakerke, Belgium). For the ICDAS-II scores, inter- and intraexaminer reproducibility was measured using kappa-Cohen statistical test. Kappa values above 0.75 denoted excellent agreement, while values between 0.40 and 0.75 indicated good agreement [13].

For each examiner, the relationships between both techniques and the histological scoring system (Downer) were assessed using the Spearman rank correlation. Data obtained from these measurements were used to calculate sensitivity and specificity at the D1 diagnostic threshold as gold standard. The use of a gold standard is a prerequisite in assessing the receiver operating characteristic (ROC) curve [14]. This analysis involves a plot of pairs of sensitivity and “1-specificity” for a given cut-off value of a diagnostic test [15]. Since this study is focusing on early detection of carious lesions, we select D1 level as diagnostic threshold. Using these sensitivity and specificity values, area under ROC curve (AUC) was carried out for each investigator and method. The performance of each method for AUC was interpreted by using the following classification: 0.50–0.60 fail, 0.60–0.70 poor, 0.70–0.80 fair, 0.80–0.90 good, and 0.90–1.0 excellent [14, 15]. The McNemar test was used to compare the sensitivity, specificity, and AUC between examiners and examinations. 

## 4. Results

A total of 18 teeth were examined with both methods by two examiners and by histology.  Table 2 shows intra- and interexaminer reproducibility analysis. The degree of intra- and interexaminer reproducibility for ACIS device was good. The weighted kappa values for intra- and interexaminer reproducibility for ICDAS-II using LPMLED were good to excellent (Table 2). 

Area under curve (AUC) values, sensitivity, and specificity of the examination methods based on D1 diagnostic threshold are presented in Table 3 for each examination. The overall AUC performance was 0.60 to 0.65 for ACIS device and 0.87 to 0.93 for ICDAS-II using LPMLED. ICDAS-II scores using LPMLED showed statistically significant higher AUC performance than ACIS device readings.

Spearman's correlation coefficients in relationship between examinations using Downer classification system are presented in Table 4. It is generally accepted that a correlation coefficient of 0.70 or above represents a strong relationship between two variables. There was a statistically significant correlation between histology and ICDAS-II scores using LPMLED.

## 5. Discussion

Occlusal surfaces account for the majority of new carious lesions, effecting both primary and permanent dentitions in children. Although occlusal surfaces are the most visited sites during clinical examination, complex occlusal anatomy and histopathology of the disease makes detection of early caries lesions difficult [16–21]. If dentistry is to move from restorative to a preventive and therapeutic based approach, early caries detection and quantification of lesions to monitor their arrest or progression over the time is essential. One of the purposes of the ICDAS-II system and ACIS device is to overcome this short fall and describe the earliest visible changes on all tooth surfaces. Clinical results of the ICDAS-II system provide an acceptable prediction of caries depth [22, 23] and scientific data for reproducibility of ICDAS-II caries detection are promising. According to Ismail et al. [24], the ICDAS-II presents good to excellent reproducibility (kappa coefficients ranged between 0.59 and 0.91). In a study where ICDAS-II codes were used in both primary and permanent teeth [25], intra- and interexaminer reproducibility values were found to be excellent (weighted kappa values > 0.82). Even when using a detailed system (ICDAS-II), there might be a degree of subjective interpretation due to perhaps visual perception and lighting problems. This is why we assessed the impact of low-powered magnification (2.5x) and LED headlight illumination using ICDAS-II criteria. Surprisingly, very little scientific research with diverging results about the influence of visual aids on caries detection has been published so far. One study showed that the use of low-powered magnification significantly improved the accuracy of examination [26], and a more recent article found that the use of magnification caused a drop in reproducibility of the ICDAS-II scores [27]. Although both examiners started to use these visual aids for the first time with this study, our results showed excellent intraexaminer (0.90–0.95) and interexaminer reproducibility (0.80) ICDAS-II using LPMLED. Our results indicate that the use of a standard criterion for visual inspection with the help of visual aids tends to increase the intra- and interexaminer agreement and makes ICDAS-II system a highly reproducible diagnostic modality for occlusal caries diagnosis.

Unfortunately there are not many published materials for the performance of ACIS device. One in vivo study demonstrated substantial agreement for both intra- and interexaminer repeatabilities of ACIS device [28]. When compared with this study, our study showed relatively low intraexaminer (0.62–0.65) and interexaminer (0.64) reproducibility. A possible explanation of this problem is the problems that both examiners encountered on the tip of the device sensor. The sensor tip easily bends after each application and this affects both the angulation and the pressure applied on tooth surface. From a clinical standpoint, care should be given not to push the sensor on tooth forcefully for the consistency of the readings.

In vitro studies usually establish the validity of a detection system by using histology as gold standard. Histological validation of caries is difficult as preparing thin sections entails tooth tissue loss, and hemisecting a tooth through the lesion may not always pass through the deepest aspect of the lesion in question. These problems arise because of the three-dimensional nature of the spread of caries dictated by the complex anatomy of the occlusal surface. A lesion may originate at one site on the surface of the tooth but spread obliquely and nonsymmetrically beneath the tooth surface. To overcome this problem and to record the deepest aspect of the lesion, we examined 1–4 sections from each tooth depending on the severity of the lesion. The worst histological score from these histological sections was recorded as reference section. Downer histology classification system [12] was used in this study to calculate sensitivity and specificity at the D1 diagnostic threshold for each examiner and examination methods. When ICDAS-II examination using LPMLED was used, the sensitivity and specificity scores for examiners were 0.87–0.90 and 0.70–0.75, respectively, at the D1 diagnostic threshold. These scores were similar with previous studies where sensitivity scores ranged from 0.69 to 0.92 and specificity scores ranged from 0.79 to 0.82 at the D1 diagnostic threshold [16, 29]. 

When ACIS device was used lower values of sensitivity (0.57–0.62) and specificity (0.68–0.70) were achieved. In a previous study where permanent teeth and a microcomputerized tomography technique for histology was used, better sensitivity (0.92) and specificity (0.92) values were recorded [30]. This difference can be explained with the anatomical variations on the occlusal surfaces of permanent and primary teeth and perhaps the difference for the histology technique.

When ICDAS-II examination using LPMLED was used, excellent AUC performance and strong correlation with histology were found by both examiners. Despite its potential, the AUC performance for ACIS on primary teeth was low. Since the previous studies showed promising results for ACIS device, a possible reason for this low performance would be the variation in the conductance of electrical impulses due to enamel thickness of primary teeth. 

To our best knowledge, this is the first study carried out using both systems on extracted primary teeth. According to our results visual aids had a remarkable positive impact on early caries detection in primary molars. A good explanation for this impact might be the increase in the depth of vision and the intensity/brightness of white light, which enhances the visibility of occlusal anatomy. 

## 6. Conclusion

Within the limitations of this in vitro study it can be concluded that the use of low-powered magnification (2.5x) and LED headlight illumination compliments ICDAS-II system in caries detection. Clinicians should keep in mind that visual aids have the potential to improve the performance of early caries detection and clinical diagnostics in children.

## Figures and Tables

**Table 1:** Criteria used in the histological examination [12].

Score	Criteria used in the Downer histological examination [12]
0	No enamel demineralisation or a narrow surface zone of opacity (edge phenomenon)
1	Enamel demineralisation limited to the outer 50% of the enamel layer
2	Demineralisation involving the inner 50% of the enamel, up to the enamel-dentine junction
3	Demineralisation involving the outer 50% of the dentine
4	Demineralisation involving the inner 50% of the dentine

**Table 2:** Intra- and interexaminer reproducibility (weighted kappa).

	AC Impedance Spectroscopy	Low powered magnification + LED
Examiner 1	0.6286	0.9057
Examiner 2	0.6572	0.9515 SE
Examiner 1 versus Examiner 2	0.6473	0.8089 SE = 0.1061

**Table 3:** Area under the ROC curve (standard error), sensitivity, and specificity at D1 diagnostic threshold.

D1 diagnostic threshold	AC Impedance Spectroscopy		Low-powered magnification + LED	
	Examiner 1	Examiner 2	Examiner 1	Examiner 2
AUC (SE)	0.60 (0.14)	0.70 (0.13)	0.93 (0.04)*	0.87 (0.09)*
Sensitivity	0.62	0.57	0.90	0.87
Specificity	0.68	0.70	0.70	0.75

AUC: area under the curve; SE: standard error.

*Statistically significant difference (*P* < 0.05).

**Table 4:** Spearman's correlation coefficients using Downer classification systems.

	Examiner 1	Examiner 2
AC Impedance Spectroscopy versus histology	0.37	0.36
Low powered magnification and LED versus histology	0.78*	0.73*

*Correlation significant at the 0.05 level.
